# Retraction: Evaluation of 3D surface scanners for skin documentation in forensic medicine: comparison of benchmark surfaces

**DOI:** 10.1186/1471-2342-8-15

**Published:** 2008-08-11

**Authors:** Wolf Schweitzer, Martin Häusler, Walter Bär, Michael Schaepman

**Affiliations:** 1 Institut für Rechtsmedizin, Universität Zürich, Zürich, Switzerland; 2 Centre for Geo-Information, Department of Environmental Sciences, Wageningen University, The Netherlands

Dr. Schweitzer submitted this article [1] to BMC Medical Imaging on the assumption that he and his co-authors had permission to use the data presented within. Since this is not the case, the authors asked for the article to be removed. Dr. Schweitzer is deeply sorry for any inconvenience this may have caused to the editorial and publishing staff. An apology is also extended to the readers and manufacturers of the scanners that were tested.

## Pre-publication history

The pre-publication history for this paper can be accessed here:


